# Predictive Equation for Basal Metabolic Rate in Normal-Weight Chinese Adults

**DOI:** 10.3390/nu15194185

**Published:** 2023-09-27

**Authors:** Xiaojing Wang, Deqian Mao, Zechao Xu, Yongjun Wang, Xiaoguang Yang, Qin Zhuo, Ying Tian, Yuping Huan, Yajie Li

**Affiliations:** 1 Key Laboratory of Trace Element Nutrition of National Health Commission (NHC), National Institute for Nutrition and Health, Chinese Center for Disease Control and Prevention, Beijing 100050, China; wangxiaojing9705@163.com (X.W.); maodq@ninh.chinacdc.cn (D.M.); wangyongjun519@163.com (Y.W.); xgyangcdc@163.com (X.Y.); 2 Beijing Chaoyang District Center for Disease Control and Prevention, Beijing 100050, China; juliexzc@163.com; 3 Department of Clinical Nutrition, The First Affiliated Hospital of Shandong First Medical University & Shandong Provincial Qianfoshan Hospital, Jinan 250014, China; 4 China-DRIs Expert Committee on Macronutrients, Beijing 100050, China; 5 Department of Nutrition and Food Hygiene, School of Public Health, Yangzhou University, Yangzhou 225009, China; tianyingjob@126.com; 6 Department of Cuisine and Nutrition, School of Food Science and Engineering, Yangzhou University, Yangzhou 225127, China; 18762302056@163.com; 7 Changzhi Medical College, Changzhi 046000, China; yjsome@163.com

**Keywords:** basal metabolic rate, Chinese adults, predictive equation, Bland–Altman plots

## Abstract

This study aimed to develop a predictive equation for basal metabolic rate (BMR) in normal-weight Chinese adults and provide a reference for establishing the national recommended dietary energy intake. A new equation for BMR was derived from a sample of 516 normal-weight Chinese adults (men = 253, women = 263), and this sample was collected from two previous studies. Furthermore, the accuracy of this new equation and eight other previous predictive equations was reviewed. The agreement and reliability were compared in terms of bias, accuracy, the intraclass correlation coefficient, and Bland–Altman plots between predictive equations. In addition, the newly developed equation was further verified using a small independent sample, which contained 41 healthy Chinese adults (men = 21, women = 20). The measured BMR (mBMR) of all participants, measured using indirect calorimetry, was 1346.2 ± 358.0 kcal/d. Thirty participants were excluded based on Cook’s distance criteria (Cook’s distance of ≥0.008). Previous equations developed by Henry, Schofield, Harris–Benedict (H-B), Yang, and Hong overestimated the BMR of healthy Chinese adults. The present equation displayed the smallest average bias (0.2 kcal/d) between the mBMR and predicted basal metabolic rate (pBMR). The limits of agreement of the present equation from Bland–Altman plots were −514.3 kcal/d and 513.9 kcal/d, which is the most narrow and balanced limit of agreement. Moreover, in the verification of the testing database, the pBMR of the new equation was not significantly different from the mBMR, and the accuracy was 75.6%. Compared with pre-existing equations, the present equation is more applicable to the prediction of BMR in healthy Chinese adults. However, further studies are required to verify the accuracy of this new equation.

## 1. Introduction

Numerous studies have demonstrated that people have increased energy intake and decreased energy expenditure due to changes in lifestyle and the way people work, such as being sedentary and consuming a high-energy diet [1,2,3]. Basal metabolic rate (BMR) accounts for 60–75% of total energy expenditure and can be defined as the minimum rate of energy expenditure in an awake, relaxed person lying on a bed in a thermoneutral environment after an overnight fast [4]. The accurate assessment of the BMR of an individual or a certain population is essential for the development of national energy requirement recommendations and patient management [5,6].

Indirect calorimetry (IC) is the gold standard for estimating BMR with a high accuracy [7]. However, stringent measurement conditions, complex operating procedures, and a high cost make it difficult to implement this method in large populations. BMR varies among individuals depending on age, sex, height, weight, fat-free mass, and physiological status [8]. Therefore, the predictive equation derived from these factors is now widely used as an alternative method for measuring BMR in order to determine the appropriate clinical treatment and nutritional management [9,10,11]. A growing number of studies have found that BMR varies considerably between ethnicities and that the predictive equations obtained from Western populations, such as Harris–Benedict, overestimate the BMR in Asian populations [12,13]. Therefore, previous researchers have explored the predictive equations suitable for the Chinese population [12,13,14,15], but they all had a small sample size, a large variability in demographic characteristics, and a lack of regional representation. In recent years, many studies have found that the relationship between BMR and weight varies in BMI-specific groups. For example, the slope of BMR and BMI in obesity is lower than in normal-weighted adults [16,17]. Therefore, BMR prediction from a weight group-specific formula is recommended for underweight subjects. Furthermore, it was also found that BMR remained stable in adulthood (20–60 years) [18]. Providing dietary guidance and treatment for metabolic health problems may be compromised and ineffective because of inaccurate BMR data [19]. Therefore, a more accurate equation should be developed for the Chinese population. Although many studies have performed BMR prediction in overweight/obese populations [20], older adults [21], and adolescents [22], only a few studies have focused on evaluating the BMR in normal-weight adults, who make up a large proportion of society’s workforce. Accordingly, this study aimed to control the confounding factors to obtain a representative predictive equation and to provide a more accurate predictive equation for BMR in healthy Chinese adults. 

## 2. Materials and Methods

### 2.1. Study Design

A training dataset was used to derive a new equation, and the BMR of participants in the training dataset was predicted using the equations progressed by Harris–Benedict [8], Schofield, Henry [23], Liu [12], Yang [13], Singapore [14], Hong [15], and AA Ganpule [24], as well as the new equation, which are listed in Table 1.

This training dataset was collected from Mao [25] and Wu’s study [26], and the participants were from six provinces in China (i.e., Beijing, Hebei, Sichuan, Heilongjiang, Shenzhen, and Hunan). Data of the participants’ sex, age, weight, and height were obtained. The BMR of the participants was measured using a cardiopulmonary function tester (Cosmed, K4b^2^, Italy) or MM3B gas analyzer (Cortex, Leipzig, Germany). Participants aged <18 years and those with a BMI of <18.5 kg/m^2^ or ≥24.0 kg/m^2^ were excluded. Finally, 516 healthy adults of a normal weight aged 18–45 years were included in this training dataset. 

In addition, a testing dataset was made, composed of 41 health adults of a normal weight, and the BMR of the participants was measured using an MM3B gas analyzer (Cortex, Leipzig, Germany). The testing dataset was used to further verify the accuracy of the new equation.

All procedures involving human subjects were approved by the National Institute for Nutrition and Health Chinese Center for Disease Control and Prevention Ethical Review Committee (Ethical approval No. 2005-09-15, No. 2006-09-12, No. 2009-02-12 and No. 2014-018). Written informed consent was obtained from all subjects.

### 2.2. The Determination of BMR

The BMR of the participants was measured using a cardiopulmonary function tester (Cosmed, K4b^2^, Italy) or MM3B gas analyzer (Cortex, Leipzig, Germany). The measurement period, the quality check, and the precision of the two devices to estimate BMR are detailed in Mao and Wu’s articles in detail. Briefly, BMR was measured in the morning when the participants, after 12 h of fasting, were awakened gently from sleeping and asked to lie down quietly. Each participant slept in a single room where the temperature was controlled between approximately 20 and 25 °C and humidity was controlled between approximately 40% and 60% on the night before the test. The BMR measurement was carried out in the early morning. Before the measurement, participants were awakened and retained a quiet, awake, and comfortable status. During this procedure, the participants could not move or speak. Females avoided being measured during their menstrual period. Prior to use, both of the IC systems were warmed up for at least 45 min and then calibrated prior to every test according to the manufacturer’s instructions. Oxygen consumption (VO_2_) and carbon dioxide production (VCO_2_) were measured for 11 min using the analyzer, and the first minute was discarded. Both of the two different IC devices use a facemask system, open-circuit spirometry systems, and breath-by-breath VO_2_ measurement methods. The two devices have been demonstrated to be reliable and show consistency of measurement between the two devices [27].

### 2.3. Statistical Analysis

Statistical analyses were conducted using SAS 9.4 and MedCalc 19.5.6 software. The mean ± standard deviation (mean ± s.d.) was used to describe the distribution of the age, height, weight, BMI, measured basal metabolic rate (mBMR), and predicted basal metabolic rate (pBMR) of the study participants. Multiple linear regression was applied to generate a new equation based on the training dataset. Regression diagnostic techniques, such as the Cook’s distance and multicollinearity diagnostics, were used to optimize the regression equation.

Paired *t*-tests were used to compare the bias among the mean pBMR calculated by nine equations (one generated equation and eight predictive equations) and the BMR measured using IC. The alpha level was adjusted by Bonferroni (*p* = 0.05/9 ≈ 0.006). The relationship between mBMR and pBMR was assessed using simple correlation analysis. In addition, to test the agreement between the IC and the predictive equations for measuring BMR, the Bland–Altman method [28] was used to assess for presence of bias and the 95% limits of agreement. The intraclass correlation coefficient (ICC) and correct classification fraction (CCF) were also calculated to evaluate the reliability of the two methods. CCF was defined as the fraction of participants whose pBMR was within 10% of the mBMR.

## 3. Results

### 3.1. Characteristic of the Training Dataset Participants

This training dataset included 516 participants from various geographic regions of China, aged 18–45 years, with a BMI of 18.5 kg/m^2^ to 24.0 kg/m^2^. In the regression diagnosis, 30 participants, who were considered to be influence points, were excluded. No significant differences were observed in the anthropometric characteristics of the participants before and after exclusion (Table 2).

### 3.2. Development of the Present Predictive Equation

Sex, weight, and height were included in the multiple linear regression analysis to establish the predictive equations. However, a significant multicollinearity was found in the collinearity diagnosis [29] for weight and height (conditional index = 102.2, Pearson’s correlation coefficient between height and weight r = 0.83, *p* < 0.001). As weight was most commonly used for estimating BMR, height was removed from the regression analysis of the equation. Subsequently, 30 influential points (5.8% of all study participants) were filtered based on the Cook’s distance criteria [30]. It is hard to re-investigate the cause of these outliers, so that 30 participants were not enrolled in the subsequent analysis. Thus, the new predictive equation was developed based on the results of the multiple linear regression analysis of the sex and weight of 486 participants. No significant difference was observed in the mBMR between the new database (1315.2 ± 306.7 kcal/d) and the original database (1346.2 ± 358.0 kcal/d) (*p* = 0.42).

### 3.3. Difference and Correlation between mBMR and pBMR

The differences and correlations between mBMR and pBMR are presented in Table 3. Paired *t*-tests showed that the Harris–Benedict (H-B), Schofield, Henry, Yang, and Hong equations significantly overestimated the mBMR, whereas the Singapore equation significantly underestimated the mBMR. No significant differences were found between the mBMR and pBMR predicted by the Liu equation, AA Ganpule equation, and the new equation. MBMR was significantly correlated with the pBMR derived from all equations. However, H-B had a minimum correlation coefficient of 0.243. The other correlation coefficients were similar, and the correlation coefficient of the present equation was 0.518.

### 3.4. Agreement between mBMR and pBMR

**Figure 1:**
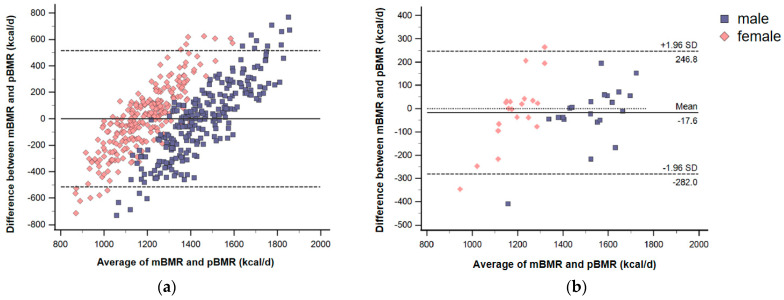
Bland–Altman plots describing the agreement between mBMR and pBMR from new equation in this study. (**a**) Description of the training dataset; (**b**) description of the testing dataset (n = 41). Delta mBMR–pBMR (kcal/d) is plotted against the average of mBMR and pBMR (kcal/d). Solid horizontal line indicates the mean difference between the two methods in kcal/day. Dashed lines depict the 95% limits of agreement (mean difference ± 1.96 SD) expressed in kcal/day. Participants were filtered by sex (pink represents female and blue represents male).

Results of the statistical analysis of the agreement between the two methods (IC and the predictive equation) are presented in Table 4. The Bland–Altman analysis showed that the means of difference and the limits of agreement for the equations from Western populations, such as the H-B, Schofield, and Henry equations, demonstrated a poor performance. The Hong equation showed the worst performance of all the equations, with a mean difference of −310.2 kcal/d, and 95% of the participants had a mean difference between −834.5 kcal/d and 214.1 kcal/d. The accuracy rates of the H-B and Hong equations were also low (25.7% and 21.8%, respectively). Both groups grossly overestimated the BMR of the participants. All of the ICCs of the H-B, Henry, and Hong equations were lower than 0.4, which indicates less agreement. The ICC and accuracy rates worked less well in all the equations. However, the present equation had the smallest mean difference (Figure 1a), where the solid horizontal line is close to 0, and the limits of agreement are relatively narrow.

### 3.5. Verification in the Testing Dataset

This testing dataset included 41 participants aged 20–25 years. BMI ranged from 18.5 kg/m ^2^ to 24.0 kg/m ^2^. Average age and BMI are listed in Table 5. Paired t-tests showed that no significant differences were found between the mBMR and pBMR predicted by the new equation. The means of mBMR and pBMR were 1349 kcal/d and 1367 kcal/d, respectively. The mBMR was significantly correlated with the pBMR, and the correlation coefficient was 0.826. The results of the Bland–Altman analysis are shown in Figure 1b, and there was a mean difference between −282.0 kcal/d and 246.8 kcal/d. Furthermore, the accuracy of this new equation in the testing dataset is 75.6% and the ICC is 0.807.

## 4. Discussion

An accurate equation for assessing the BMR is critical for determining energy requirements based on a factorial approach, which is calculated by multiplying physical activity level by the BMR value. The present BMR for adults in the dietary energy of Chinese DRIs is expressed in BMR/kg body weight, which will overestimate the BMR of heavy individuals and underestimate that of light individuals [11]. Accordingly, it is imperative to establish an accurate BMR-estimated equation that considers body weight and body composition to revise the recommended dietary energy intake of Chinese adults. The present study developed a more representative equation for Chinese adults of a normal weight.

To date, most of the predictive equations that have been widely used for BMR assessment are derived from Western countries, such as the H-B, Henry, and Schofield equations (Table 1). Although they have been used to obtain BMR values in clinical and scientific studies worldwide [31,32], they grossly overestimate the BMR of Asian individuals [33]. The present study demonstrated that the H-B equation was not suitable for the study participants as it had the lowest correlation coefficient (0.243) and penultimate accuracy (25.7%). A previous study has also provided evidence that the H-B equation make erroneous estimates of BMR for Chinese populations [31]. This might be because this equation was derived 100 years ago and is limited compared to relative technologies. Ethnicity is commonly believed to be an important factor contributing to the bias in the predictive equation. Although thousands of participants were included in the Schofield database, 45% of them were Italians [34], and these individuals had a significantly higher BMR compared to those of other races. Therefore, it is not difficult to conclude that the Schofield equation overestimated the participants’ BMRs in this study. Although Henry et al. attempted to change the disproportionate database [23], it still overestimated the BMRs by an average of 98.6 kcal/d in this study. These differences may be attributed to the lack of Chinese participants in their database [23].

In recent years, researchers have been developing predictive equations suitable for the Chinese population, such as the Liu, Yang, Singapore, and Hong equations. The results of the predictive equations used on participants from limited regions, such as Taipei, Chengdu, and Singapore, were not representative of populations from mainland China. As shown in Table 3, the equation-estimated BMR means obtained using the Yang, Hong, and Singapore equations were all significantly different to the mBMR. This is supposedly because age and physical activity level affect the metabolism, and the mechanisms by which different ages and physical activity levels affect the BMR have not yet been determined exactly. It is supposed that physical activity level and age may influence fat-free mass, and fat-free mass can influence the BMR. Current studies have found that age and BMR do not always show a linear correlation, with BMR remaining essentially constant from 20 to 60 years of age and decreasing after the age of 60 years [18]. Liu’ s participants, aged between 20 and 78 years, and the participants in this study, aged 60–78 years, might result in heterogeneity. Furthermore, elevated physical activity levels will cause changes in muscle mass and cell volume, increasing the BMR [35]. Therefore, it is not surprising that the Hong equation overestimated the BMR by 85.0%, with Bland–Altman plots yielding limits of agreement of −638.0 kcal/d and −77.3 kcal/d in almost three-fifths of study participants (182 of 315, 57.8%) who were youths who had high chronic physical activity levels [15]. The results of ICC analysis for the nine equations showed that six equations have relatively good repeatability (>0.400), while the H-B, Henry, and Hong equations have lower repeatability.

Compared with previous studies, this study has several positive features worth noting. First, to our knowledge, this study has the largest sample size for predicting BMR in normal-weight adults living in mainland China. The participants have different occupations, such as students, farmers, and government employees, and are from six provinces in China. Second, this study strictly controlled for variables of the participants’ characteristics, like age and weight, and was dedicated to developing a predictive equation with good generalizability among healthy Chinese adults of a normal weight. The results of this study showed that the mean pBMR of the present equation was not different from the mean mBMR. The equation developed in this study had the smallest mean of difference and the smallest width of the limits of agreement, with 95% of the bias being included in the limits of agreement. Thirdly, an additionally testing dataset containing 41 healthy adults was used to test the applicability of these equations. The result demonstrated that the new equation has a good applicability and accuracy in this population.

However, this study has several limitations. First, the participants in this study were gathered from different databases, and BMR was obtained using two different open-circuit IC methods. This could be the reason for the small R-square in the present equation (R^2^ = 0.269) and the apparent bias seen in Figure 1. Although BMR measurements using open-circuit calorimetry show little difference due to the variations in the types of equipment used [27], assurance that the same environment with the same instrument and under the same measurement conditions was provided may yield a more reliable mBMR. Second, this equation is limited by the lack of participants aged 45–60 years, whose utility should be assessed by testing the older Chinese population. Similarly, the testing dataset was relatively small and was not representative due to limitations in terms of age and geography for assessing the validity of the new equation, and more datasets are required to test this equation.

Owing to the numerous known limitations of IC, the prediction of individual BMR using readily available predictors such as height, weight, and sex is still the preferred method for clinical and nutritional practitioners. A survey found that 80% of nutrition workers were accustomed to using a predictive equation to obtain BMR [36].

## 5. Conclusions

In conclusion, the present equation has a relatively better consistency and accuracy compared with previous equations. Hence, the BMR of healthy Chinese participants is best predicted using the present equation. This equation is essential for proposing the national estimated energy requirement for normal-weight adults. The utility of this equation will be further enhanced if it is validated on an ongoing basis across different regions and in older populations.

## Figures and Tables

**Table 1:** Equations for BMR.

Equation Source	Sex	Age (Years)	Equation
Harris–Benedict (kcal/d)	Males	21–70	66.473 + 5.003 H + 13.752 W − 6.775 A
	Females	21–70	655.096 + 1.850 H + 9.563 W − 4.676 A
Schofield (kcal/d)	Males	18–30	15.057 W + 692.2
	Females	18–30	14.818 W + 486.6
	Males	30–60	11.472 W + 873.1
	Females	30–60	8.126 W + 845.6
Henry (kcal/d)	Males	18–30	16.0 W + 545
	Females	18–30	13.1 W + 558
	Males	30–60	14.2 W + 593
	Females	30–60	9.74 W + 694
Liu (kcal/d)	Both	20–78	13.88 W + 4.16 H − 3.43 A − 112.40 S + 54.34
Yang (kJ/d)	Both	18–45	89 W + 600 S + 277
Hong (kcal/d)	Both	18–67	13.9 W − 5.39 A − 247 S + 1102
Singapore (kJ/d)	Both	21–69	52.6 W – 196 S + 2974
AA Ganpule (kJ/d)	Both	≥20	48.1 W + 23.4 H – 13.8 A – 54.73 S + 123.8
New equation (kcal/d)	Both	18–45	14.52 W – 155.88 S + 565.79

W: weight (kg); H: height (cm); A: age (years); S: sex (men = 0, women = 1).

**Table 2:** Demographic and anthropometric variables of the participants.

	Male		*p*	Female		*p*	Total		*p*
	Database 1 (*n* = 253)	Database 2 (*n* = 236)		Database 1 (*n* = 263)	Database 2 (*n* = 250)		Database 1 (*n* = 516)	Database 2 (*n* = 486)	
Age (years)	26.9 ± 7.1	26.9 ± 7.2	0.98	27.0 ± 7.7	26.5 ± 7.4	0.53	26.9 ± 7.4	26.7 ± 7.3	0.62
Height (cm)	169.8 ± 6.0	169.4 ± 5.7	0.40	158.9 ± 5.4	158.9 ± 7.2	0.92	164.3 ± 7.9	164.0 ± 7.6	0.44
Weight (kg)	61.7 ± 6.6	61.3 ± 6.1	0.30	51.2 ± 5.2	53.2 ± 5.1	0.93	57.4 ± 7.3	57.1 ± 6.9	0.82
BMI (kg/m^2^)	21.3 ± 1.5	21.3 ± 1.4	0.86	21.0 ± 1.5	21.1 ± 1.5	0.86	21.2 ± 1.5	21.2 ± 1.5	0.10

Note. Database 1: original database; Database 2: new database, in which the influential points were filtered.

**Table 3:** Difference and correlation between the measured BMR and predicted BMR.

Equation Source	Mean ± s.d. (kcal/d)	Correlation Coefficient (r)
Measured BMR (n = 486)	1315 ± 307	-
Predicted BMR from		
Harris–Benedict	1579 ± 98 *	0.243
Schofield	1438 ± 183 *	0.519
Henry	1414 ± 164 *	0.518
Liu	1326 ± 169	0.511
Yang	1351 ± 198 *	0.515
Hong	1625 ± 201 *	0.510
Singapore	1283 ± 165 *	0.516
AA Ganpule	1321 ± 171	0.511
New equation	1315 ± 159	0.518

Note. * *p* < 0.006

**Table 4:** Agreement between measured BMR and predicted BMR (n = 486).

Equation	Mean of Difference (kcal/d)	Limits of Agreement (kcal/d)	Under Estimation (%)	Over Estimation (%)	Accuracy (%)	ICC (95%CI)
Harris–Benedict	−263.6	−848.6 and 321.5	7.4	66.9	25.7	0.085 (−0.023–0.191)
Schofield	−122.5	−638.2 and 393.2	13.2	48.4	38.5	0.409 (0.256–0.529)
Henry	−98.6	−613.0 and 415.7	14.4	44.4	41.2	0.399 (0.283–0.498)
Liu	−10.9	−528.2 and 506.4	25.7	31.3	43.0	0.432 (0.357–0.502)
Yang	−35.7	−556.8 and 485.4	22.2	34.8	43.0	0.465 (0.392–0.532)
Hong	−310.2	−834.5 and 214.1	3.5	74.7	21.8	0.273 (−0.068–0.529)
Singapore	32.8	−482.5 and 548.0	33.5	28.0	38.5	0.428 (0.352–0.498)
AA Ganpule	−5.7	−523.1 and 511.6	27.0	31.7	41.4	0.436 (0.361–0.505)
New equation	−0.2	−514.3 and 513.9	27.4	31.5	41.2	0.424 (0.348–0.494)

**Table 5:** Demographic and anthropometric variables of the validation dataset participants.

	Male (n = 21)	Female (n = 20)
Age (years)	21.4 ± 1.2	21.0 ± 1.5
Height (cm)	176.2 ± 5.5	163.2 ± 4.3
Weight (kg)	66.6 ± 7.5	53.9 ± 3.9
BMI (kg/m^2^)	21.4 ± 1.6	20.2 ± 1.3

## Data Availability

Data available on request due to ethical restrictions.
